# Excision of the Head of the Femur

**Published:** 1861-06

**Authors:** 


### NOTES OF SURGICAL CASES.

By E. ANDREWS, M. D.,

PROFESSOR OF SURGERY IN THE MEDICAL DEPARTMENT OF LIND UNIVERSITY, ETC.

### SURGICAL WARDS, MERCY HOSPITAL.

CASE 1st.—*Excision of the Head of the Femur.*

M. M., aged ten years, was attacked a year ago by an inflammation of the left hip joint, which seemed to be rheumatic in its character. Being skilfully treated by her attendant—Dr. Fox, of Lyons, Ill., she apparently made a perfect recovery. About six months afterwards, she was attacked with a malignant diphtheria, through which she was carried also by the skill of Dr. Fox. The diphtheria, however, left her in a very enfeebled and cachectic condition. The hip joint took on a new inflammation, and one of different character from the previous attack. The case soon assumed all the appearances of hip disease, and progressed with great rapidity. In a short time pus was effused, and a discharge of half a pint every day, soon reduced the patient to the verge of fatal exhaustion.

Under these circumstances, the immediate removal of the carious bone was decided to afford the only chance of life. For this purpose a mixture of chloroform and ether was given, to anæsthesia, and the diseased part laid open by a single incision of four inches parallel to the shaft of the femur. On introducing the finger, the acetabulum was found to have entirely lost all definite boundaries by absorption of its rim. It was converted into a broad shallow fossa, and was half filled with granulations. I find this state of the cavity in a large portion of all the cases of hip disease which come under my care for operation. The head of the femur was carious, denuded of cartilage, and lay with its rough burr-like surface in contact with the flesh on all sides. With the chain saw I removed the head and neck of the bone, and closed the wound with a single adhesive strap. There was no material hæmorrhage, and the patient awoke from sleep without manifesting a particle of shock from the operation.

One beneficial result of the removal of the diseased bone was an immediate diminution of the suppuration, and of the pain upon moving the limb. The next day the patient was put upon the free use of the mur. tinct. of iron as a tonic, and as a prophylactic against traumatic erysipelas, and now—ten days after the operation, is doing remarkably well.

